# The SESAMEEG package: a probabilistic tool for source localization and uncertainty quantification in M/EEG

**DOI:** 10.3389/fnhum.2024.1359753

**Published:** 2024-03-13

**Authors:** Gianvittorio Luria, Alessandro Viani, Annalisa Pascarella, Harald Bornfleth, Sara Sommariva, Alberto Sorrentino

**Affiliations:** ^1^Bayesian Estimation for Engineering Solutions srl, Genoa, Italy; ^2^Department of Mathematics, University of Genoa, Genoa, Italy; ^3^CNR, Institute for Applied Mathematics “Mauro Picone”, Rome, Italy; ^4^BESA GmbH, Gräfelfing, Germany

**Keywords:** Bayesian inference, inverse problems, MEG, EEG, open-source software, MATLAB, Python

## Abstract

Source localization from M/EEG data is a fundamental step in many analysis pipelines, including those aiming at clinical applications such as the pre-surgical evaluation in epilepsy. Among the many available source localization algorithms, SESAME (SEquential SemiAnalytic Montecarlo Estimator) is a Bayesian method that distinguishes itself for several good reasons: it is highly accurate in localizing focal sources with comparably little sensitivity to input parameters; it allows the quantification of the uncertainty of the reconstructed source(s); it accepts user-defined *a priori* high- and low-probability search regions in input; it can localize the generators of neural oscillations in the frequency domain. Both a Python and a MATLAB implementation of SESAME are available as open-source packages under the name of SESAMEEG and are well integrated with the main software packages used by the M/EEG community; moreover, the algorithm is part of the commercial software BESA Research (from version 7.0 onwards). While SESAMEEG is arguably simpler to use than other source modeling methods, it has a much richer output that deserves to be described thoroughly. In this article, after a gentle mathematical introduction to the algorithm, we provide a complete description of the available output and show several use cases on experimental M/EEG data.

## 1 Introduction

The electromagnetic signals at the scalp produced by neural currents in the brain are the most direct consequences of brain electrical activity and can be non–invasively measured by means of MagnetoEncephaloGraphy (Hämäläinen et al., 1993) (MEG) and ElectroEncephaloGraphy (Baillet et al., 2001) (EEG). Remarkably, M/EEG recordings can be acquired at the outstanding sampling rate of the order of the millisecond (Gratta et al., 2001), thus opening the door to the study of the dynamics of neural processes in a wide variety of conditions, both normal (Sorrentino et al., 2006; Brookes et al., 2011) and pathological (Stoffers et al., 2007; Stam et al., 2009; Uda et al., 2012; Luria et al., 2020), with very high precision in time.

Mapping the activity of known sources in the brain to the corresponding M/EEG signals is called the *forward* problem (Pursiainen et al., 2011; Vorwerk et al., 2016). This is a well-posed problem which is solved by giving a parametric representation of the sources and by modeling how the electromagnetic field propagates through the brain compartments. Two main source models have been proposed in the literature so far: the Distributed Source (DS) model and the Equivalent Current Dipole (ECD) model. While in the former the neural current is assumed to be a continuous vector field inside the brain volume, the latter model assumes instead the whole brain activity underlying the M/EEG measurements to occur only in a small number of clusters of thousands of synchronously activated pyramidal cortical neurons. In this setting, each cluster is represented by a point source, called ECD, and the whole primary current distribution is approximated by the superposition of a given number of ECDs. Notably, the ECD model is currently the standard approach in clinical applications of MEG, such as the pre-surgical localization of epileptic spikes, and the only one recommended by the American Clinical Magnetoencephalography Society (Bagic et al., 2011; Carrette and Stefan, 2019). In order to model the propagation of the electromagnetic field through the head, it is crucial to exploit the information about the physical and geometrical properties of the head, which can be gathered from high resolution anatomical Magnetic Resonance Imaging (MRI). Then, discretization of the differential equations governing the electromagnetic fields can be done using Boundary Element Methods (BEM) or Finite Element Methods (FEM); however, BEM can only be used to model homogeneous and isotropic conductivity, which is clearly a too simplistic model; FEM, on the other hand, allow to model inhomogeneous and anisotropic conductivity, but accurate estimates of the spatially-varying conductivity tensor are typically difficult to obtain. As a consequence, despite being a well-posed problem, the forward solution is typically affected by modeling errors as well as numerical inaccuracies.

The capability of solving the forward problem leads to the possibility of inferring the location of the generators of brain activity from M/EEG data, which in turn is called the *inverse* problem. This last is ill-posed, since it suffers from the non-uniqueness of the solution, and exhibits a high sensitivity to noise. As a consequence, data need to be complemented with anatomical and physiological prior knowledge, thus sacrificing the exact match between the recorded and the reconstructed electromagnetic field.

The vast majority of available methods for source localization provide a single, unique “best” reconstruction of neural activity from a given dataset, with no quantification of the degree of reliability of the reconstruction itself, nor any clue about the existence of alternative solutions. However, ill-posedness implies that it is impossible to restore the neural generators exactly; hence, when solving the inverse problem we should not content ourselves with a single best estimate, and should instead answer the following questions too: are there *other* potential solutions? how certain are we of the single estimate provided? Answering these questions is difficult: in order to do it, it is necessary to characterize the probability distribution of the neural current conditioned on the measured data, i.e. the posterior distribution of the Bayesian approach.

In a set of publications (Sorrentino et al., 2013, 2014; Luria et al., 2019; Viani et al., 2021, 2022) we proposed a fully Bayesian algorithm, based on the ECD source model and belonging to the class of Sequential Monte Carlo (SMC) samplers (Del Moral et al., 2006), to solve the M/EEG localization problem. This inverse solver, called SEquential Semi-Analytic Montecarlo Estimator (SESAME), is able to sample the whole posterior distribution for the multi-dipole configuration, thereby providing multiple alternative solutions, each with an associated quantification of its reliability. In a couple of recent studies SESAME has been shown to score very well in terms of localization accuracy when compared to wMNE and MUSIC in Luria et al. (2020) and to a larger set of inverse solvers in Pascarella et al. (2023), while also being particularly stable with respect to input parameters.

In the present paper we present both a Python and a MATLAB open-source implementation of SESAME, under the name of the SESAMEEG package. The main idea behind SESAMEEG is to provide a user-friendly tool that can be used out-of-the-box by the general audience, but also lets the experienced user the possibility of providing different kinds of prior knowledge about the problem. Moreover, to facilitate the entire analysis pipeline, SESAMEEG is well integrated with the most popular open source M/EEG software and SESAME is also implemented in the commercial CE-marked software package BESA Research.

The paper is organized as follows. In Sections 2.1.1–2.1.2 we provide a gentle mathematical introduction to the ECD model and to the Bayesian approach to source modeling. In Section 2.1.3 we discuss the impact of input (hyper)parameters on the output of SESAME, described in Section 2.1.5 A very brief summary of the computations behind SESAME is provided in Section 2.1.4 and the factors affecting the computational cost of the algorithm are discussed in 2.1.6. The SESAMEEG package is described in Section 2.2 and we then proceed with exemplar analysis of experimental datasets: an MEG dataset in Section 3.1 and an EEG dataset in Section 3.2. Finally, in Section 4 we discuss the current and future work.

## 2 Method

### 2.1 SESAME: a Bayesian algorithm for M/EEG source modeling

#### 2.1.1 The source model

Source localization of M/EEG data is typically based on the following model:


(1) $$\begin{array}{l} y\left(t\right)=\overset{N}{\underset{i=1}{\sum }}G\left(r_{i}\right)q_{i}\left(t\right)+\epsilon \left(t\right) \end{array}$$

where:

- *y*(*t*) is the measured data at time *t*, modeled as the superposition of contributions of different sources;
- *G*(*r*_*i*_) is the lead field corresponding to a point source located at *r*_*i*_;
- *q*_*i*_(*t*) represents the neural current at location *r*_*i*_ at time *t*;
- ϵ(*t*) is (Gaussian) noise, accounting for measurements noise as well as forward modeling errors.

In (1) the sum over *i* represents the additive contributions of sources located at different points *r*_*i*_ in a given discretized source space. For the sake of simplicity, we henceforth omit the time dependence of all variables.

We underline that it is perhaps not common to include forward modeling errors in the additive noise term ϵ; however, we reckon it is important to do so because even exact measurements cannot be explained exactly in a real environment, due to the unavoidable approximations in the forward model. The use of a Gaussian distribution to model this contribution might be questionable: so far this choice is mainly based on practical reasons and lack of better knowledge; the same model was used in other studies, e.g., Rimpiläinen et al. (2019).

In distributed models, source locations {_*r*_*i*_}*i* = 1, …, *N*_ are assumed to be known *a priori* and the number *N* of distinct source locations is typically large (~ 10, 000). The only unknowns would be the values {*q*_*i*_}_*i* = 1, …, *N*_: once these have been estimated, one can localize brain activity as the points corresponding to maximum values of *q*_*i*_. The number of unknowns is large (~ 3 × 10, 000) but data depend linearly on the unknowns.

In the multi-dipole model, the same Equation (1) is used with the following differences: the number *N* is now unknown but small (lower than 10); source locations are also unknown. Therefore the total number of unknown parameters to be estimated is much fewer than the corresponding number in the distributed model, but data depend non-linearly on *N* and {_*r*_*i*_}*i* = 1, …, *N*_, which makes the problem harder.

In our formulation, we adopt a multi-dipole model for brain activity. We assume that, within the considered time interval, both the number and location of the active sources remain fixed; the only parameter that has a time dependence is the intensity of each active source.

#### 2.1.2 The Bayesian model

The starting point of Bayesian methods is a set of conceptual tenets (Kaipio and Somersalo, 2007; Pascarella and Sorrentino, 2011):

- probability is used to quantify uncertainty about any variable involved in the problem;
- because of the previous item, all variables are considered random variables; this does not imply that such variables are *random* in an ontological sense, but just that our knowledge of their values is imperfect, and such imperfection can be represented with a probability distribution;
- the mathematical rule to combine *a priori* information with information coming from the data is Bayes rule.

SESAME is a Bayesian inference tool applied to a multi-dipole model: it aims at approximating the posterior distribution of the number of sources *N*, the source locations *R* = {*r*_*i*_}_*i* = 1, …, *N*_ and the source strengths *Q* = {*q*_*i*_}_*i* = 1, …, *N*_, given the data


$$\begin{array}{l} p\left(N,R,Q|y\right)=\frac{p\left(y|N,R,Q\right)\;p\left(N,R,Q\right)}{p\left(y\right)} \end{array}$$

where

- *p*(*N, R, Q*|*y*) at the left hand side is the posterior distribution;
- the likelihood function *p*(*y*|*N, R, Q*) is set to be Gaussian of standard deviation σ_ϵ_, accounting for the presence of noise in the data as well as errors in the forward model;
- the second term at the right hand side is the prior *p*(*N, R, Q*);
- the denominator is a normalizing constant.

In SESAME we make the further assumption that the unknowns are *a priori* independent *p*(*N, R, Q*) = *p*(*N*)*p*(*R*)*p*(*Q*) and set a Gaussian prior on the source strengths $p\left(Q\right)=N\left(0,\sigma_{q}\right)$; combined with the Gaussian likelihood, this leads to a conditionally linear Gaussian model for source strengths. As a consequence, in the following standard splitting of the posterior


$$\begin{array}{l} p\left(N,R,Q|y\right)=p\left(Q|N,R,y\right)\;p\left(N,R|y\right) \end{array}$$

the first bit at the right hand side can be computed analytically, while the second bit is approximated with a SMC sampler algorithm (Del Moral et al., 2006), briefly described in Section 2.1.4.

#### 2.1.3 Hyper-parameters

It is important to remark that the posterior distribution depends on the two hyper-parameters mentioned above, namely the standard deviation σ_ϵ_ of the Gaussian likelihood and the standard deviation σ_*q*_ of the Gaussian prior on the dipole strength. Here we briefly explain how to deal with them.

We start by considering the Gaussian prior on the dipole strength, with its corresponding standard deviation σ_*q*_. It is important to remark that a Gaussian prior is a fairly strong prior, that forces the unknown to be of the same order of magnitude as the standard deviation. In principle, this fact can be even used to our own advantage: in Luria et al. (2019) we showed that SESAME can be used to mimick distributed sources by setting a small σ_*q*_, that forces small dipoles and produces more widespread reconstructions. On the other hand, for a standard analysis with a purely dipolar model the dependence of the solution on the value of σ_*q*_ is actually annoying, but can be strongly reduced by introducing a *hyper-prior*, i.e. a prior on the hyper-parameter σ_*q*_. This was done originally in Viani et al. (2021), where we presented an updated model in which the hyper-parameter σ_*q*_ is considered unknown, and treated as an additional parameter, i.e. sampled from the hyper-prior and then updated in the SMC steps. In order to provide as little information as possible on the order of magnitude of the sources, we chose to use a log-uniform hyper-prior in the interval $\left[\sigma_{q}^{min},10^{3}\sigma_{q}^{min}\right]$, where $\sigma_{q}^{min}$ is chosen based on the order of magnitude of the data and of the lead field. We have shown that the introduction of the hyper-prior makes the estimated configuration stable across over three orders of magnitude of the (hyper-)hyper-parameter. In the SESAMEEG package, the user can choose whether to use the hyper-prior and basically ignore the problem of setting σ_*q*_, or else to use the value estimated from the lead field and the data, or else to set a value manually.

The standard deviation of the Gaussian likelihood σ_ϵ_ currently represents the main hyper-parameter of SESAME. Understanding its role is key for an effective use of the algorithm. Roughly speaking, the value σ_ϵ_ represents a threshold below which the discrepancy between the measured data and the data produced by the solution can be ignored: if this threshold is set low, then the algorithm will do its best to reproduce the data accurately; to this aim, it will likely produce solutions with larger number of dipoles (which also takes a lot of time). If the threshold is set high, then the algorithm will produce simple solutions that fit the data only approximately. As already pointed out in Section 2.1.2, the role of this hyper-parameter in the Bayesian model is to take into account both noise in the data and uncertainties/errors in the forward model. Therefore finding a good value is not always straightforward, and we are working on removing the dependence on this hyper-parameter too (Viani et al., 2023). In the open-source SESAMEEG packages, σ_ϵ_ is by default estimated as the 20% of the peak of the signal, as a rule-of-thumb assessment of the two contributions of measurement noise and forward modeling error; the experienced user is allowed to change the value of this hyper-parameter to their liking.

#### 2.1.4 The SMC sampler algorithm

At the core of SESAME is a Sequential Monte Carlo sampler that approximates *p*(*N, R*|*y*) with a weighted set of candidate solutions, termed *particles*, {(_*N*_*i*_, *R*_*i*_), *w*_*i*_}*i* = 1, …, *I*_, where *w*_*i*_ represents the weight of the *i*-th candidate solution (*N*_*i*_, *R*_*i*_).

In this subsection we provide a very brief summary of the computations behind SESAME: for more details we invite the reader to consult (Sommariva and Sorrentino, 2014; Sorrentino et al., 2014; Viani et al., 2021), where the mathematical model and the algorithm have been thoroughly described.

The recipe is as follows. An initial set of particles is drawn from the prior distribution, then the following steps are repeated until convergence:

1. [MCMC step] each particle is randomly perturbed within a neighborhood; also, dipoles can be added or removed from the particle; the perturbation can be accepted or rejected, based on whether the perturbed version fits the data better than the original one;
2. [Reweighting] particle weights are updated based on an importance sampling rule,
3. [Resampling] the particle set may undergo a *resampling* procedure, i.e.: particles with low weights are discarded and particles with large weights are duplicated.

The final set of particles is then used to produce estimates from the posterior distribution.

#### 2.1.5 SESAME output

Once the posterior has been approximated, SESAME can provide answers to the following questions: how many sources are there? What are most probable source locations? How certain are we about the source locations?

In particular, standard SESAME output encompasses:

- the posterior probability of different number of sources *p*(*N* = *i*|*y*) for *i* = 0, 1, … ; this can be visualized e.g. as a pie chart, as is done below;
- the posterior probability of source locations *p*(*R*|*y*), typically visualized as a probability map on the brain surface or in the brain volume; here, a highly focused map indicates low uncertainty on the estimated source locations; on the contrary, a widespread map indicates high uncertainty;
- the most probable source locations, identified by first estimating the number of sources as the number $\hat{N}$ by maximizing *P*(*N*|*y*), and then identifying $\hat{N}$ peaks in the posterior probability $p\left(R|y,\hat{N}\right)$;
- the source time courses of the most probable sources.

At times, the posterior probability of the number of sources will assign comparable probabilities to distinct models: for example, it can happen that 60% probability is assigned to a one-dipole model and 40% probability is assigned to a two-dipole model. In these cases, SESAME provides both alternative solutions, so that the user can evaluate which one is more likely to be correct, based on additional information they might have.

#### 2.1.6 SESAME computational cost

While the specific implementation, outlined in the next Section, has an impact on the computational cost of the algorithm, few basic facts are common to all implementations:

- the vast majority of the computational cost is due to the Monte Carlo sampling of *p*(*N, R*|*y*), while the subsequent calculation of the source time courses according to *p*(*Q*|*N, R, y*) has a comparably negligible cost;
- the computational cost of the SMC sampler depends on the complexity of the posterior distribution, and in particular grows non-linearly with the estimated number of sources;
- such computational cost is linear in the number of particles.

### 2.2 The SESAMEEG packages

The SESAME algorithm has been implemented into two distinct open–source packages, one coded in Python and one coded in MATLAB, under the collective name of SESAMEEG; a commercial version is also available as a part of the BESA Research 7.0+ software. The software is platform independent and has been tested on Windows, macOS and Linux. Apart from being able to be used as a standalone software, SESAMEEG is well integrated within the most popular open–source packages for analyzing human neurophysiological data: Brainstorm (Tadel et al., 2011), MNE–Python (Gramfort et al., 2013a), FieldTrip (Oostenveld et al., 2011) and Zeffiro Interface (He et al., 2020). Such integration has the virtue of letting the user perform all the analysis pipeline steps—such as data pre-processing and visualization—within the same toolbox.

**Figure 1:**
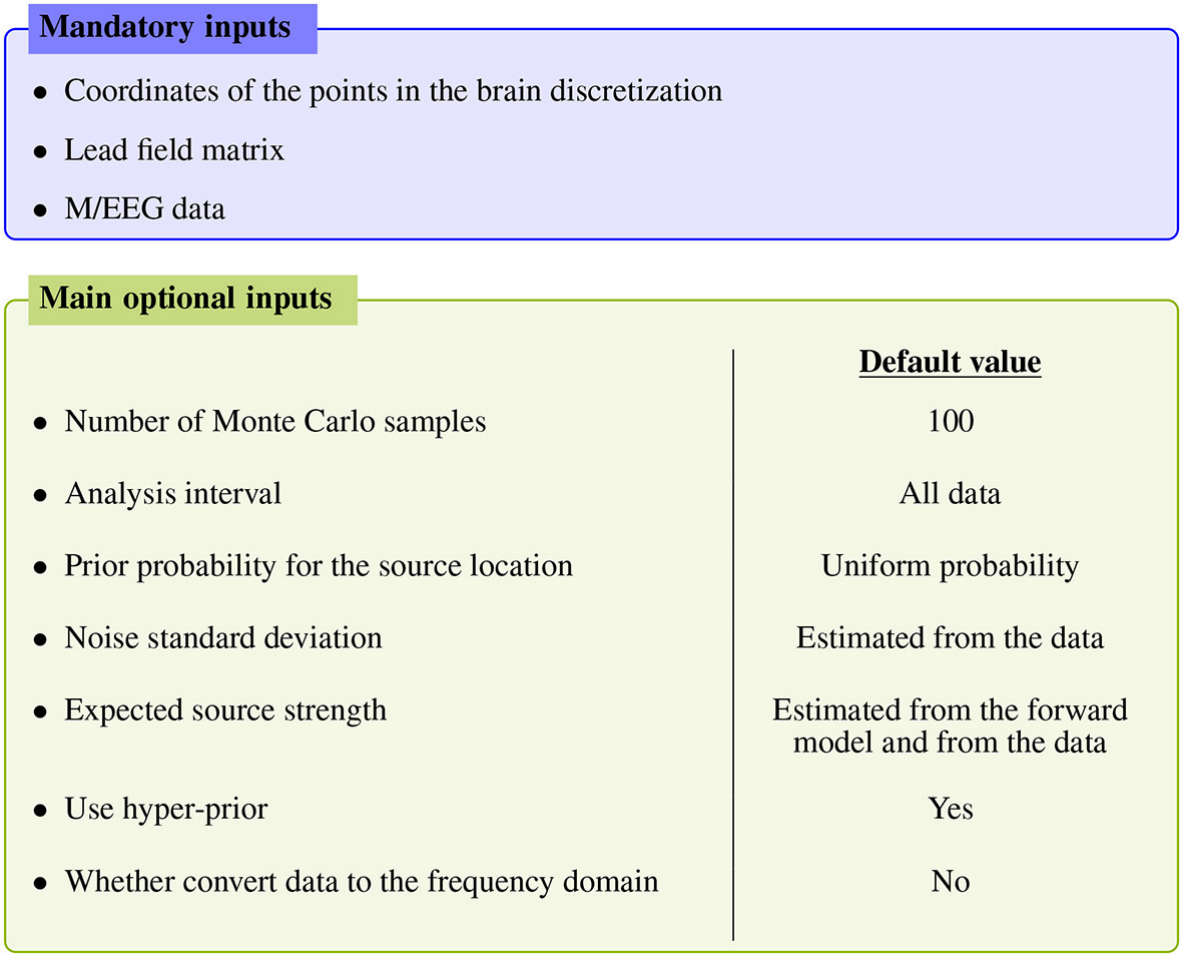
Main inputs of the SESAME algorithm. Apart from the mandatory inputs listed in the top blue box, all other inputs have an automatically estimated default value.

Figure 1 summarizes the main inputs of the algorithm; for more advanced settings the reader is referred to the API documentation, as detailed below.

The mandatory inputs are the forward solution (namely the source space and the lead field matrix) and the M/EEG data. SESAMEEG can run in different analysis scenarios: the source space can be both cortically constrained and volumetric, and the source orientations in the forward solution can be both free and orthogonal to the surface of the cortex.

Each of the remaining inputs has its own default value, which has been engineered to let SESAMEEG perform well out–of–the–box in most common scenarios. However, a high degree of customization is available to experienced users.

As described in Section 2.1.5, the output of SESAME is an empirical posterior distribution for a variable number of sources and for their parameters.

From this distribution, maximum a posteriori estimates are computed and—conditioned on the estimated number of sources—a cortical probability map is worked out, which quantifies for each voxel the posterior probability of containing a dipolar source.

SESAMEEG can visualize SESAME outputs in several different ways, which vary according to the software environment and which may also depend on the inputs: as an example, whenever a volumetric source space is used and a MRI image such as T1 is available, it becomes feasible to visualize source estimates overlaid on MRI and to morph estimates to a template brain for group analysis.

#### 2.2.1 SESAMEEG Python

The Python package SESAMEEG is available at the Python Package Index (PyPI) repository (https://pypi.org/project/sesameeg/) and distributed under a Berkeley Software Distribution (BSD) 3-Clause “New” or “Revised” License. The source code is available at the GitHub repository (https://github.com/pybees/sesameeg) while the documentation can be found at https://pybees.github.io/sesameeg/ and comes with an example gallery in which all most common use cases are illustrated.

The code is object oriented and its core functionalities are implemented in the class Sesame. When working in the MNE–Python framework the latter class has to be instantiated by means of the function mne.prepare_sesame; otherwise, if SESAMEEG is used as a standalone software, it has to be instantiated directly.

Calling the method apply_sesame on the Sesame instance then applies SESAME on the given M/EEG data and computes point estimates from the posterior distribution.

SESAME output can be visualized in multiple ways by means of the following built-in methods:

- plot_source_number plots the posterior probability of the number of sources *p*(*N* = *i*|*y*) as either a pie chart or a bar plot;
- plot_source_amplitudes plots the amplitude of the estimated sources as function of time;
- plot_sources plots the posterior probability of source locations *p*(*R*|*y*) and the estimated sources. By default, these quantities are visualized on the cortical surface or superimposed on the MRI image when working within the MNE–Python framework, and as a PyVista (Sullivan and Kaszynski, 2019) PolyData object otherwise.

The entire source model analysis can be saved into and loaded from Hierarchical Data Format (HDF) files by means of the built-in methods save_h5 and io.read_h5.

Regarding the software architecture, SESAMEEG consists of the modules sesame.py, emp_pdf.py, particles.py and dipoles.py, and also comprises several subpackages:

- sesameeg.io implements functionality to save and load SESAMEEG output;
- sesameeg.viz implements several functions to visualize SESAMEEG output;
- sesameeg.mne implements functionality to interface with MNE–Python objects;
- sesameeg.metrics implements the metrics Goodness of Fit (GOF), Optimal subpattern assignment (OSPA) (Schuhmacher et al., 2008), Map Localization Discrepancy (MLD) and Spatial Dispersion (SD) (Luria et al., 2020). GOF provides information on how well the reconstructed electromagnetic field fits the measured data, while SD quantifies the spatial dispersion of each cortical map and thus the uncertainty of the reconstruction. By default, GOF and SD are evaluated and printed at the end of each run of SESAME. All the four metrics help in quantifying the performance of SESAME whenever the ground truth is known.
- sesameeg.utils implements a number of utility functions. These include estimate_noise_std, which estimates the standard deviation σ_ϵ_ as the 20% of the analyzed signal peak and estimate_dip_mom_std, which estimates the hyper-parameter σ_*q*_ from both the lead field and the data.

#### 2.2.2 SESAMEEG MATLAB

The Matlab version of SESAMEEG is available at https://github.com/pybees/sesameeg_MATLAB. The documentation, which comes with an example script, can be found at https://pybees.github.io/sesameeg_MATLAB/.

The code is function oriented: SESAME is run by calling the inverse_SESAME function, taking as input the lead field matrix, the data matrix and the source space matrix as well as a configuration structure containing optional configuration parameters; the output of inverse_SESAME is a structure containing analysis parameters and representations of the posterior probability distribution; the essential information can be visualized by the inverse_SESAME_viewer function which shows both the posterior probability distribution for the number of sources and the cortical probability map.

**Figure 2:**
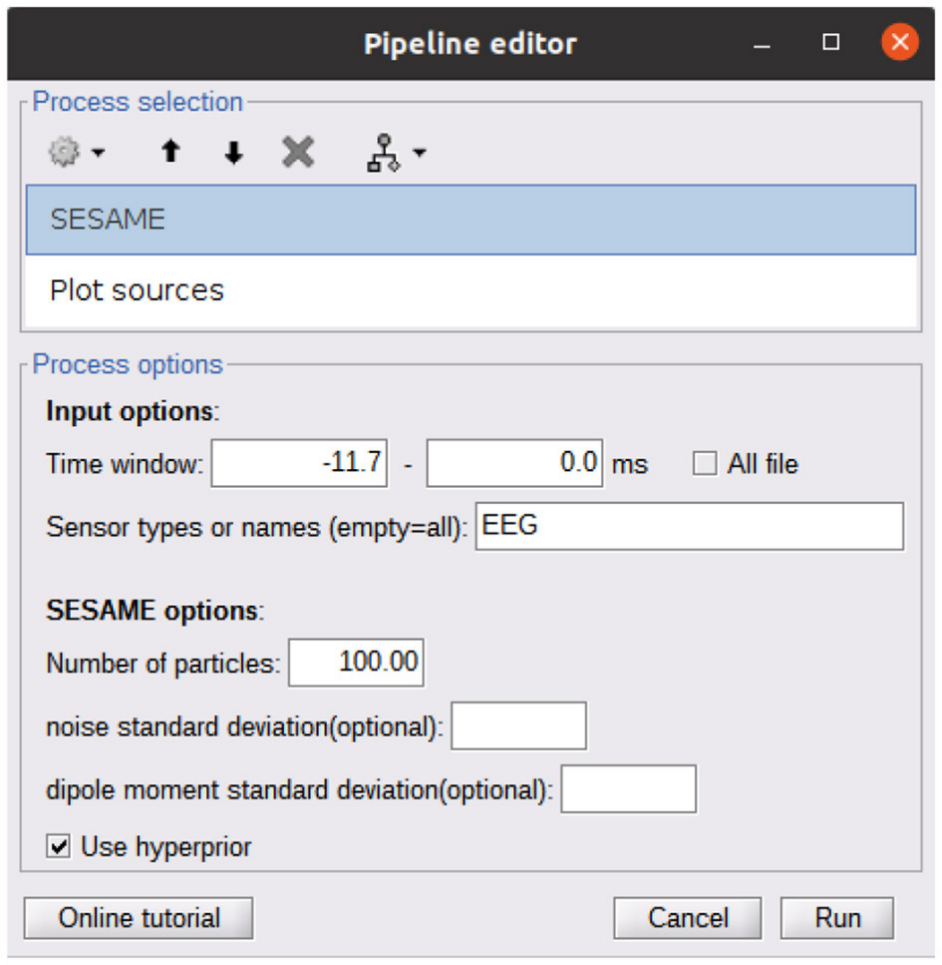
Pipeline editor GUI of Brainstorm calling the SESAME algorithm. The first process (SESAME) runs the algorithm in the selected time window, with the optional input parameters. The output of SESAME may be the input of the Plot Sources process which computes dipole estimates from the posterior distribution.

A Brainstorm plugin is also available at https://github.com/pybees/sesameeg_MATLAB/tree/main/Brainstorm in the form of the two scripts process_sesame and process_posterior: the former runs SESAME with the inputs that have been set in the Pipeline editor GUI (Figure 2); the latter takes the posterior distribution (which is the output of process_sesame) in input and computes point estimates.

As detailed in the Brainstorm tutorial on creating new processes (https://neuroimage.usc.edu/brainstorm/Tutorials/TutUserProcess), the provided scripts must be copied into the Brainstorm user folder in order to make SESAMEEG available in the pipeline editor menus.

#### 2.2.3 Commercial version: BESA

**Figure 3:**
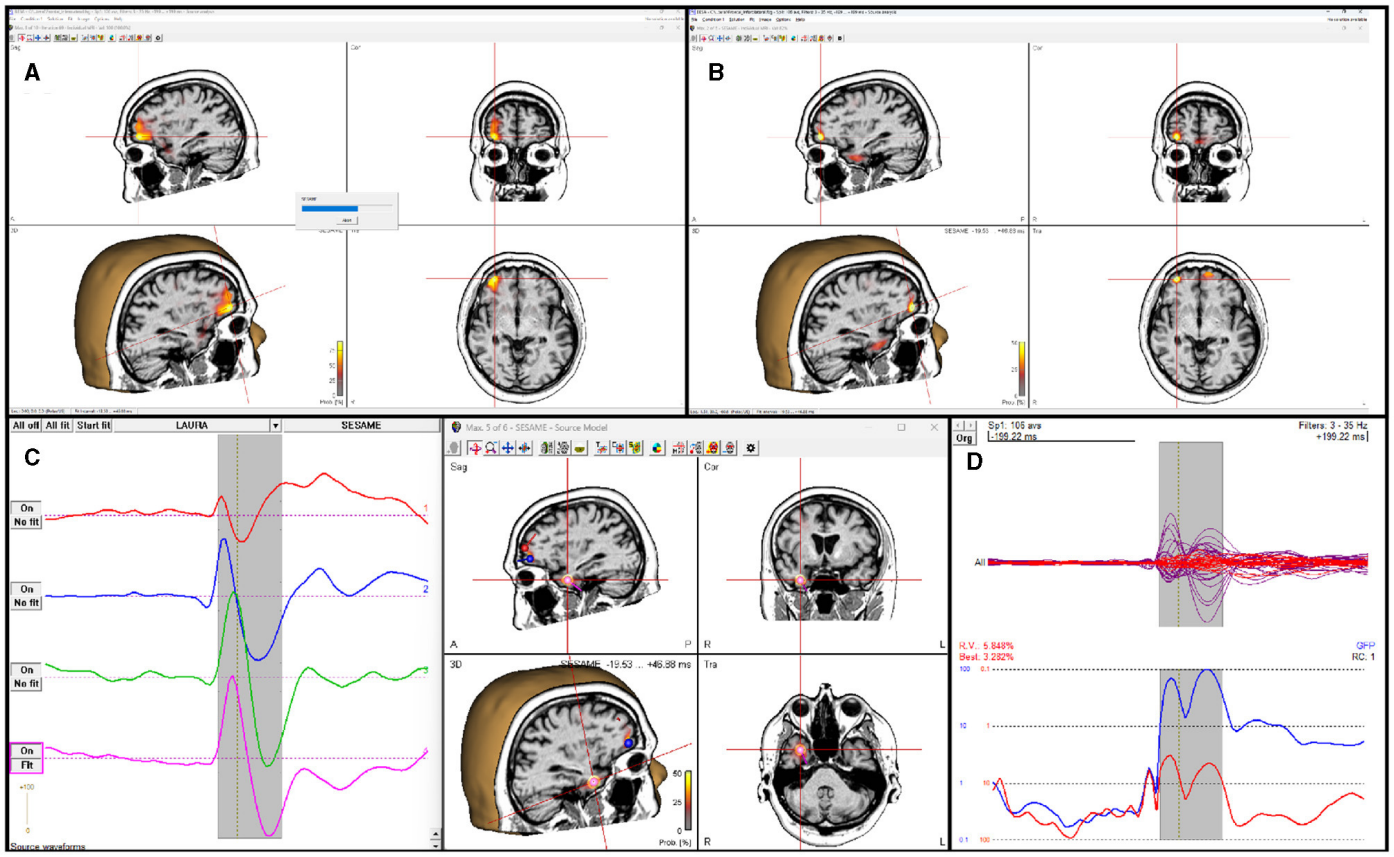
SESAME implementation in the BESA software applied to an averaged EEG segment of inter-ictal epileptic discharges. **(A)** SESAME runs through several iterations, converging to the most likely posterior map. **(B)** After completion, the user can browse through the maxima. **(C)** Sources can be seeded from maxima to examine the temporal activation pattern. **(D)** display of model, residual, and global field power after the SESAME-informed dipole solution was created.

SESAME is also implemented in the commercial CE-marked software package BESA Research version 7.0 and higher. While not being substantially different from its open-source counterparts, SESAME in BESA has the added value of being part of a complete, user friendly software for EEG and MEG data analysis. The user can analyze any data segment of EEG, MEG, or combined M/EEG data. Unlike in the open-source packages, the baseline interval is used to estimate the noise variance. Like in the open-source packages, parameters for noise and signal estimation, as well as hyper-prior usage can be adjusted by the user; spatially non-uniform priors can be set additionally, e.g., by reading in other modality data like fMRI or by running a different distributed source reconstruction method prior to invoking the SESAME algorithm. The posterior probability map is displayed in a 3D viewer. The user can browse through detected maxima in the map, and seed discrete sources from those, e.g. to determine the precise temporal activation pattern. The computation time depends on the complexity of the probability distribution. Figure 3 shows the application to an averaged EEG segment of inter-ictal epileptic discharges that had several activation foci. For this data set, computation with default parameters (using hyper-priors, perform 50 iterations) took 15 seconds on a Windows laptop with Intel Core i9 processor (2.4 GHz) and 8 cores.

## 3 Results

The present Section showcases the application of SESAMEEG in source modeling analyses from M/EEG experimental datasets. The main focus of this Section is on the presentation and interpretation of SESAME output, particularly how this is affected by the choice of the noise standard deviation parameter. For examples concerning the benefits of using of non-uniform spatial priors we refer to Viani et al. (2022), while for an example of application in the frequency domain we refer to Luria et al. (2019).

### 3.1 MEG experimental data

**Figure 4:**
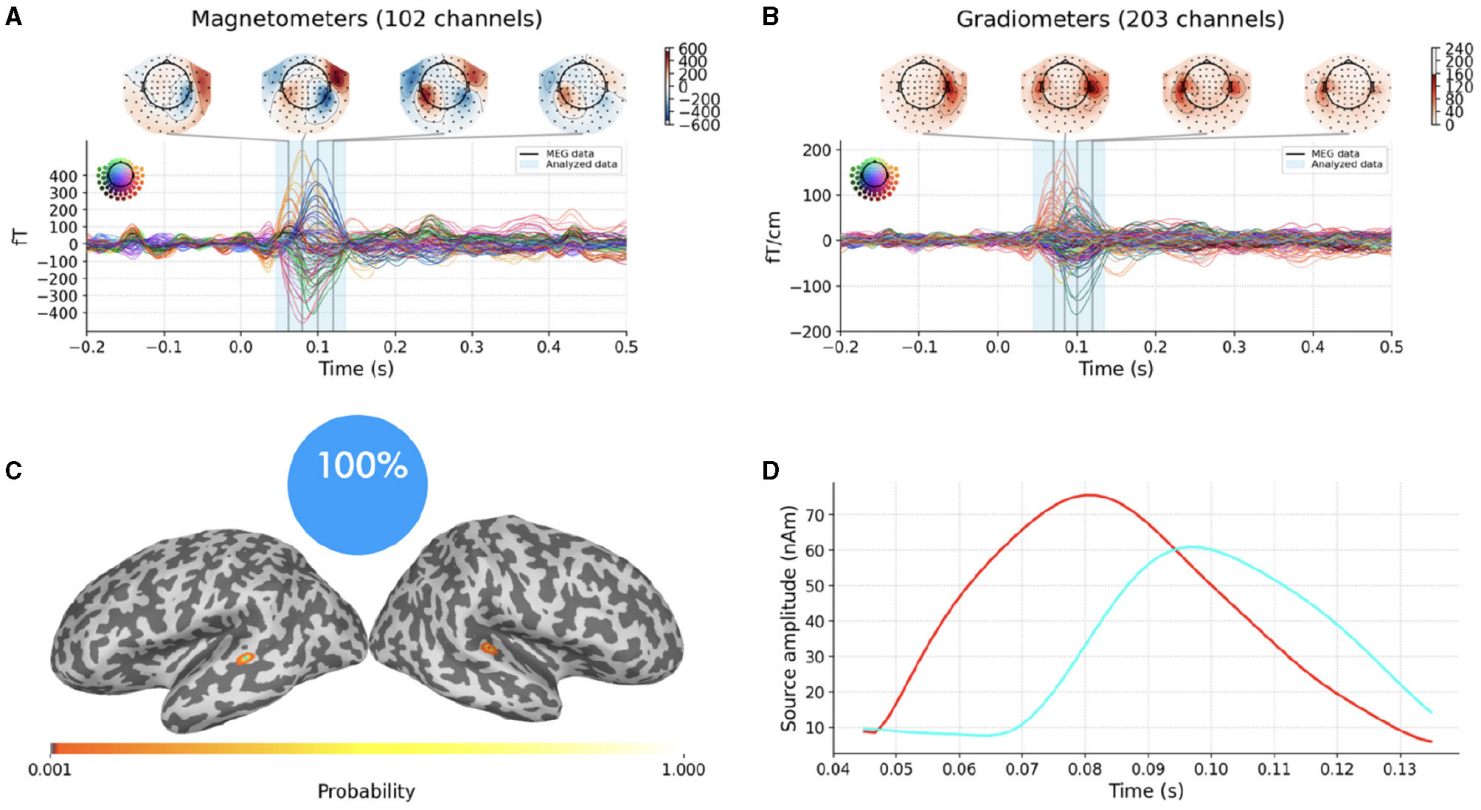
Top row: average evoked response to auditory stimuli presented to the left ear as recorded by MEG magnetometers **(A)** and gradiometers **(B)**; the vertical blue translucent rectangle denotes the time window from 45ms to 135ms analyzed by SESAME. Bottom row: Reconstructed source configuration. **(C)** shows a pie chart indicating 100% probability of the 2-dipole configuration, and the cortical probability map on an inflated brain, with colored dots representing the estimated dipole locations; preserving the same color code, the estimated source amplitude time courses are plotted in **(D)**.

Figures 4A, B portray experimental MEG data consisting in the average evoked response to auditory stimuli presented to the left ear. The description of the entire experiment can be found in Gramfort et al. (2013a,b) and will not be repeated here. Data and the forward solution are freely available in the sample open dataset which comes with the MNE-Python package.

In this Section, we exploit these data to conduct a threefold analysis with SESAMEEG Python: we first show how SESAME reconstructs the brain activity when default values are used as input parameters; then we explore how the choice of the noise standard deviation influences the obtained solution in terms of both the number and the location of the estimated dipoles; finally we present an example in which SESAME finds two alternative scenarios with non-negligible probabilities.

We extract the topographies from 45ms to 135ms around the M100 peak and we perform the source localization running SESAMEEG with its default values (see Figure 1).

As shown in Figures 4C, D, SESAME identifies, with full probability, two active dipoles as the generators of the measured field. Figure 4C depicts the cortical probability map on an inflated brain, with colored dots representing the estimated dipole locations, one in the right auditory cortex and the other contralateral very near to the auditory cortex. The posterior distribution looks sharply peaked around the estimated loci, which holds the information of a very small uncertainty in the reconstruction. We stress the fact that this map is distinct from an intensity map, as it is solely associated with the probability of the source locations. Figure 4D shows the estimated source amplitude time courses, with the same color code as in Figure 4C: the source located in the right hemisphere activates before the other, with a peak to peak latency difference between the cortices for the M100 activity that is quantified in 17ms. The estimated source configuration is therefore fairly in line with the literature (Kaiser et al., 2000; Gramfort et al., 2013b).

The whole script can be found in the documentation example gallery. For this data set, computation with the default parameters took 79 seconds on a Linux laptop with Intel Core i7 processor (3.3 GHz) and 8 cores.

**Figure 5:**
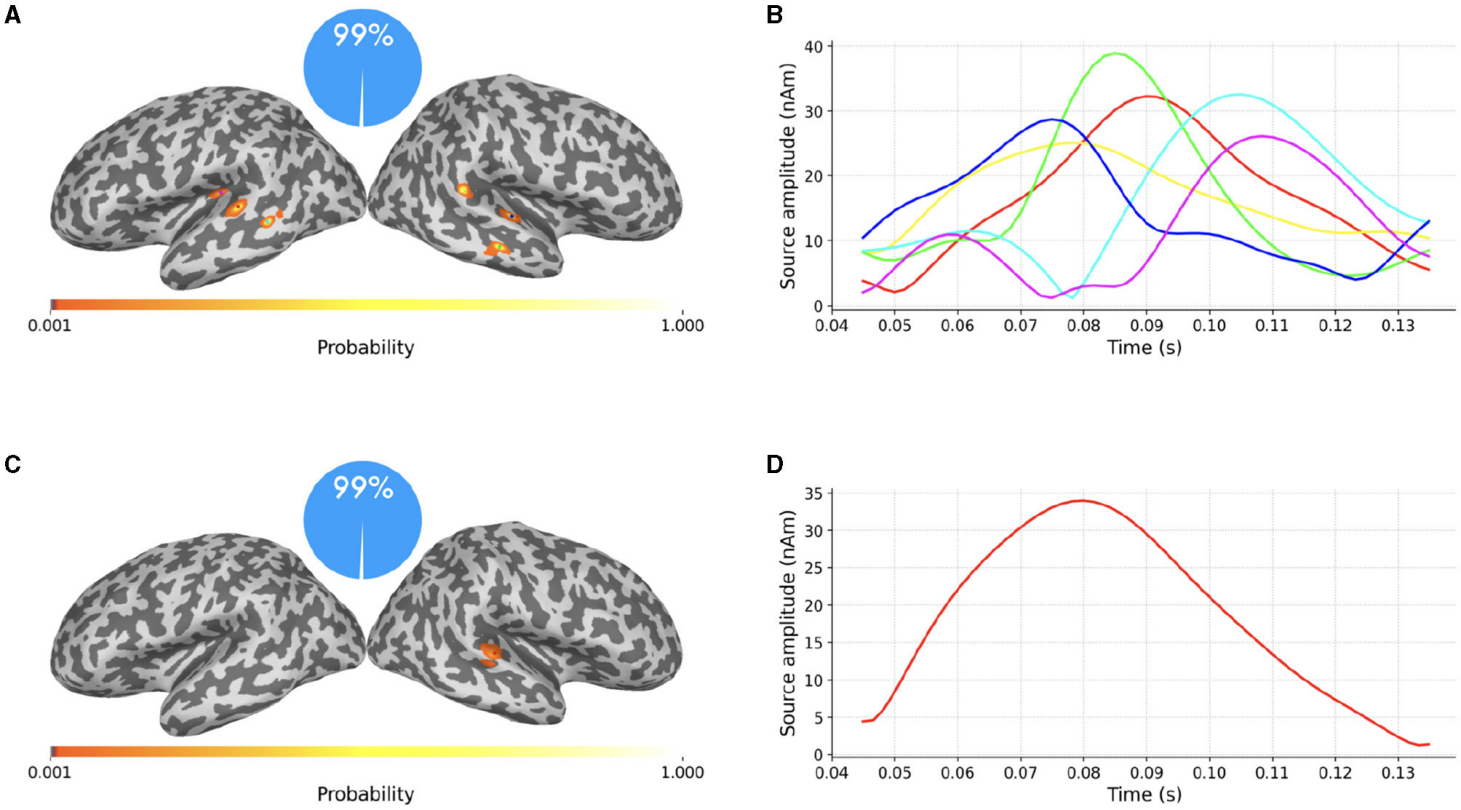
**(A–D)** SESAME source modeling results from the same data portrayed in Figure 4, with different values of *σ*_*ϵ*_. Top row: Parameter value underestimated as the 6.5% of the maximum measured magnetic field. Bottom row: Parameter value overestimated as the 65% of the data peak. Left column: cortical probability maps, with colored dots representing the estimated dipole locations. Right column: the estimated source amplitudes.

We now want to show the impact of the noise standard deviation parameter on the solution, as discussed in Section 2.1.3. To do so, we modify the analysis setting by explicitly underestimating and overestimating the parameter value with respect to SESAMEEG's default. We present the results in Figure 5.

In the top row the parameter value has been underestimated as the 6.5% of the maximum measured magnetic field. The estimated number of sources is six, with negligible probability assigned to other configurations. This result is in line with Section 2.1.3: when underestimating the noise standard deviation, SESAME has to introduce additional sources in order to explain finer details of the data. In this case, each of the two sources of Figure 4 is practically split into three components.

In the bottom row the parameter value has been overestimated as the 65% of the data peak. The estimated number of sources is one, again with negligible probability assigned to other configurations. This result is in line with Section 2.1.3 too: as lesser fit is required with the data, the estimated solution is simpler, the location is more uncertain and the estimated source time course weaker. Of the two sources of Figure 4 only the stronger one has survived.

**Figure 6:**
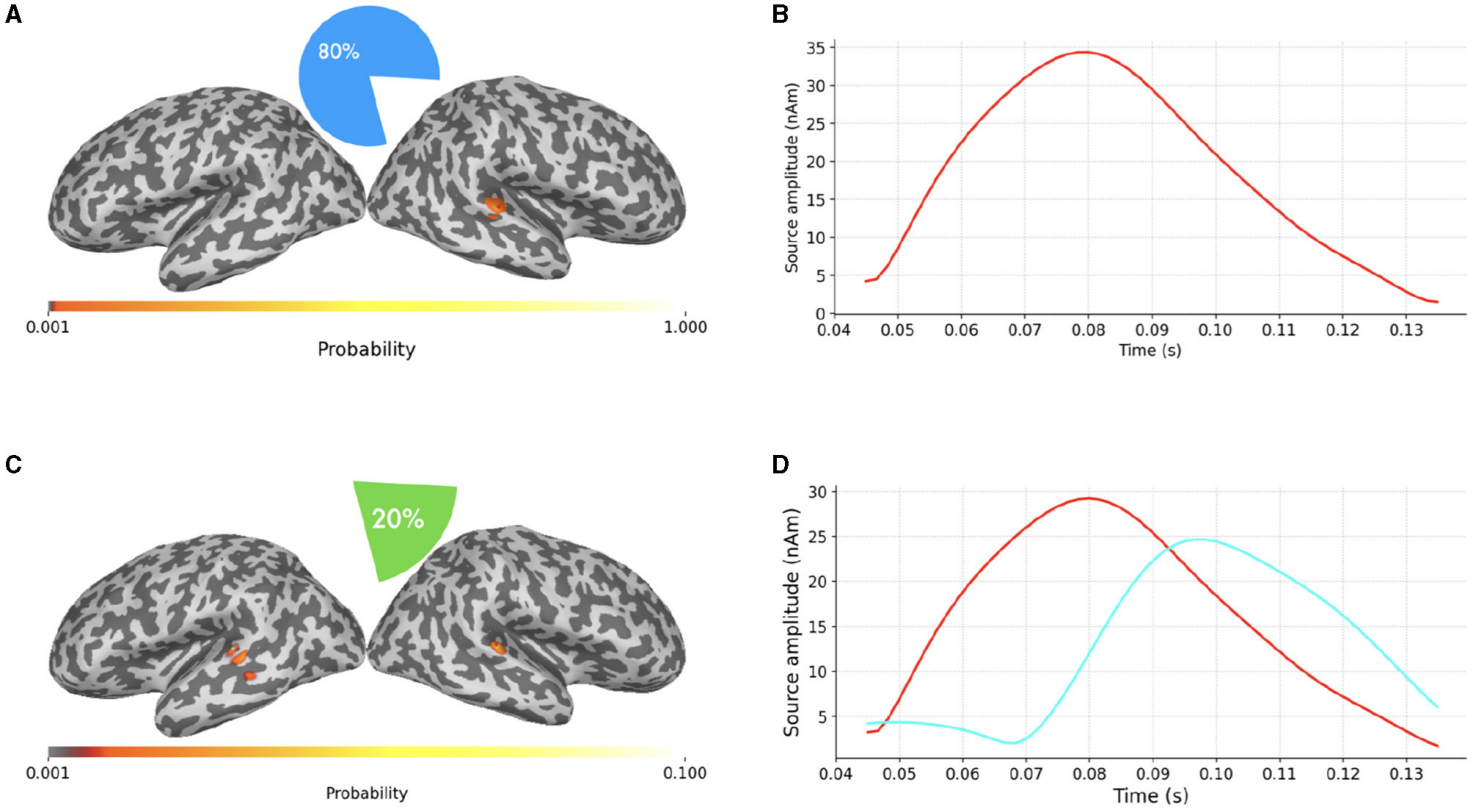
**(A–D)** Alternative SESAME solutions from the same data portrayed in Figure 4, with σ_ϵ_ set to the 64% of the data peak.

In Figure 6 we finally present a case where SESAME provides two alternative solutions with non-negligible probabilities. This output was obtained by setting the noise standard deviation as the 64% of the data peak, i.e., slightly smaller than the value used in Figures 5C, D. We happen to fall in a borderland in which both the one dipole configuration and the two dipoles configuration can possibly explain the measured field: the most probable solution clearly resembles that of Figure 5; however, with a 20% of probability, SESAME consider an alternative scenario more similar to that of Figure 4, even if the spatial localization is more uncertain in this case.

We stress the fact that being able to automatically provide information about the existence of alternative solutions and characterize their relative probabilities is an asset of SESAME which, to the best of our knowledge, is not provided by other inverse solvers that typically limit their output only to the most probable source configuration.

### 3.2 EEG experimental data

**Figure 7:**
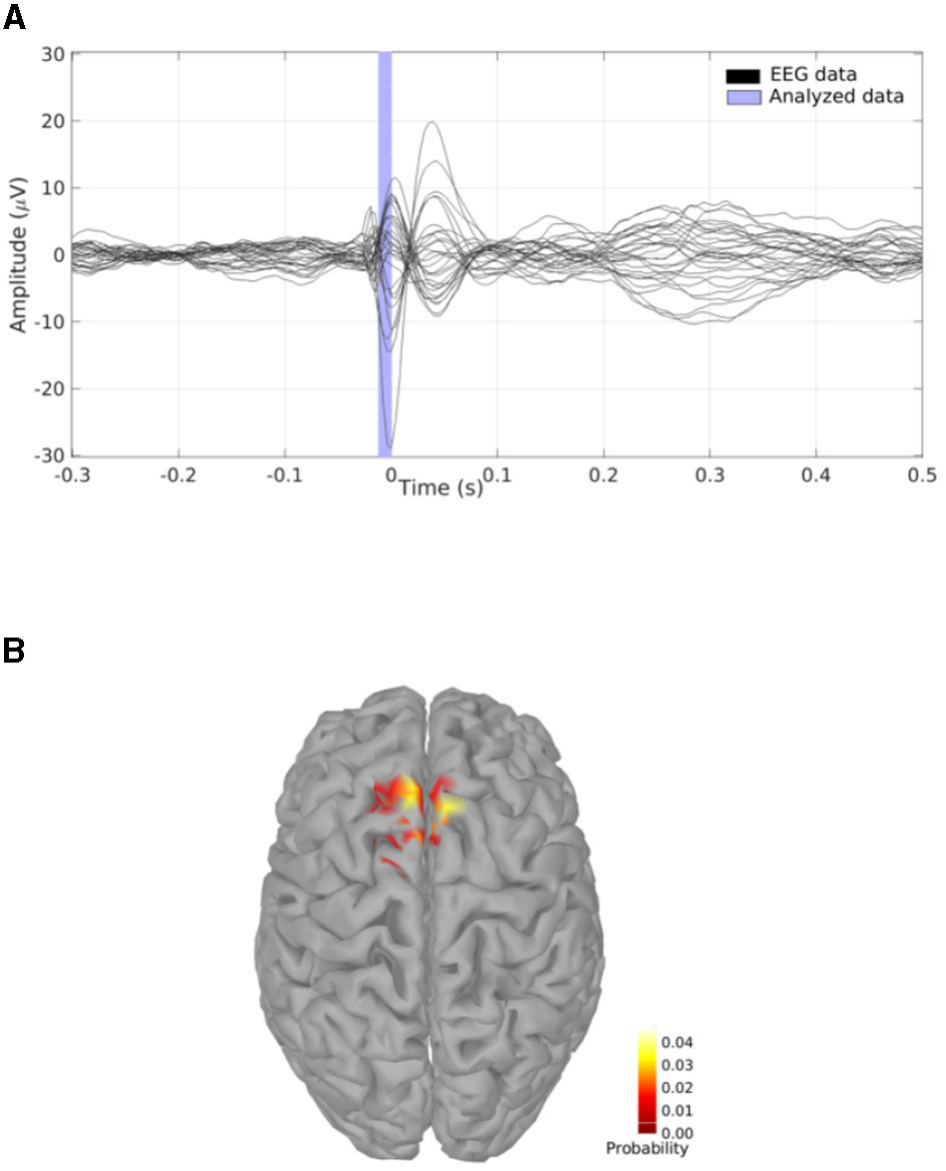
**(A)** EEG experimental data consisting in the average of multiple IEDs acquired in a patient who suffered from focal epilepsy; the vertical blue translucent rectangle denotes the time window from -11ms to 0ms analyzed by SESAME. **(B)** Cortical probability map which indicates that the irritative zone is probably contained within the superior frontal gyrus.

Figure 7A shows the average of multiple Interictal Epileptiform Discharges (IED) as recorded by a 128 channels EEG and acquired in a patient who suffered from focal epilepsy. Data are part of a Brainstorm tutorial dataset (https://neuroimage.usc.edu/brainstorm/Tutorials/Epilepsy) and we refer the reader therein for a thorough clinical description. For application of SESAME we built a BEM forward model using OpenMEEG (Gramfort et al., 2011) with three compartments and standard conductivities (scalp 1, skull 0.0125, brain 1).

We perform a source modeling analysis by means of the Brainstorm plugin of SESAMEEG. Referring to the Pipeline editor GUI depicted in Figure 2, we select the time window from -11ms to 0ms, we leave the noise standard deviation field empty so that SESAMEEG automatically estimates the parameter value as the 20% of the analyzed signal peak and we run the SESAME process using 100 Monte Carlo samples.

The resulting cortical probability map shown in Figure 7B indicates that SESAME localizes the irritative zone in the superior frontal gyrus with the estimated epileptic focus in the left hemisphere. This agrees with the clinical history of the patient who, after invasive monitoring of the supposed epileptogenic zone (Dümpelmann et al., 2012), underwent a left frontal resection which led to an Engel 1A postsurgical outcome with a follow-up of 5 years.

For this data set, computation with the default parameters took 27 seconds on a Linux laptop with Intel Core i7 processor (3.0 GHz) and 8 cores.

## 4 Discussion

Amongst available methods for source localization from M/EEG data, SESAME represents an *unicum*: on the one hand, it outperforms the majority of other localization methods in terms of reconstruction accuracy with focal sources, as shown recently (Pascarella et al., 2023); on the other hand, to the best of our knowledge, it features the unique capability of quantifying the degree of confidence of the estimated source configuration, and to provide multiple alternative scenarios whenever the data are ambiguous.

In this paper we presented SESAMEEG, a set of software packages written in different languages and easily integrated in most commonly used software analysis pipelines. In Section 3 we showed that SESAMEEG provides good reconstructions of neural activity from both MEG and EEG data when used with the default parameters. The aim of SESAMEEG is therefore to make the benefits of Bayesian source modeling of M/EEG data available to the largest possible audience.

A long way has been gone but there is still just as much to go.

To begin with, the dipolar model SESAME is based on clearly limits the applicability of the method to experimental conditions in which the involved sources are highly focused. We are currently working at a generalization of the method that encompasses the source extent among the unknown parameters to be estimated. Successful work in this direction would have the additional benefit of enabling a quantification of the extent of the source, with its associated uncertainty.

A second key point in the future development of SESAME concerns a more detailed modeling of the forward model errors. While these are currently accounted for as an zero-mean additive component, more can be done along the lines suggested e.g., in Rimpiläinen et al. (2019). Better modeling of this component would lead to more accurate source reconstruction as well as better uncertainty quantification.

Finally, as the whole Monte Carlo procedure underlying SESAME can be a bit heavy particularly when the number of sources is large, implementation of the code in a parallel environment exploiting GPUs should be pursued.

## Data availability statement

Publicly available datasets were analyzed in this study. This data can be found here: https://mne.tools/stable/generated/mne.datasets.sample.data_path.html; https://neuroimage.usc.edu/brainstorm/Tutorials/Epilepsy.

## Ethics statement

The studies involving humans were approved by the Corresponding Ethics Committee. The studies were conducted in accordance with the local legislation and institutional requirements. Written informed consent for participation was not required from the participants or the participants' legal guardians/next of kin in accordance with the national legislation and institutional requirements.

## Author contributions

GL: Conceptualization, Methodology, Software, Writing – original draft, Data curation, Investigation, Writing – review & editing. AV: Conceptualization, Investigation, Methodology, Software, Writing – original draft, Writing – review & editing. AP: Conceptualization, Investigation, Software, Writing – original draft, Methodology, Writing – review & editing. HB: Software, Writing – review & editing. SS: Software, Writing – review & editing, Conceptualization. AS: Software, Conceptualization, Methodology, Writing – original draft, Writing – review & editing.
